# Microbiome and metabolome association network analysis identifies *Clostridium_sensu_stricto_1* as a stronger keystone genus candidate than *Bifidobacterium* in the gut of common marmosets

**DOI:** 10.1128/msystems.00214-25

**Published:** 2025-07-07

**Authors:** Jordan B. Hernandez, Shivdeep S. Hayer, Sophie Alvarez, Anne Fischer, Haley R. Hassenstab, Katherine Cooper, Zahraa W. Alsafwani, Andrew K. Benson, Mallory J. Suhr Van Haute, Jacques Izard, Hyun-Seob Song, Jonathan B. Clayton

**Affiliations:** 1 Department of Biology, University of Nebraska Omaha 169231, Omaha, Nebraska, USA; 2 Nebraska Food for Health Center, University of Nebraska-Lincoln, Lincoln, Nebraska, USA; 3 Callitrichid Research Center, University of Nebraska Omaha, Omaha, Nebraska, USA; 4 Department of Genetics, Cell Biology and Anatomy, University of Nebraska Medical Center 12284, Omaha, Nebraska, USA; 5 Proteomics and Metabolomics Facility, Nebraska Center for Biotechnology, University of Nebraska-Lincoln, Lincoln, Nebraska, USA; 6 School of Interdisciplinary Informatics, College of Information Science and Technology, University of Nebraska Omaha, Omaha, Nebraska, USA; 7 Department of Food Science and Technology, University of Nebraska-Lincoln 14719, Lincoln, Nebraska, USA; 8 Fred and Pamela Buffet Cancer Center, University of Nebraska Medical Center 12284, Omaha, Nebraska, USA; 9 Frederick F. Paustian IBD Center, University of Nebraska Medical Center 12284, Omaha, Nebraska, USA; 10 Department of Internal Medicine, University of Nebraska Medical Center 12284, Omaha, Nebraska, USA; 11 Department of Biological Systems Engineering, University of Nebraska-Lincoln, Lincoln, Nebraska, USA; 12 Department of Pathology and Microbiology, University of Nebraska Medical Center, Omaha, Nebraska, USA; 13 Primate Microbiome Project, University of Nebraska-Lincoln 14719, Lincoln, Nebraska, USA; The University of Maine, Orono, Maine, USA

**Keywords:** marmoset, gut microbiome, gut metabolome, bioinformatics, multi-omics, network analysis, microbial ecology, amplicon sequencing, non-human primate

## Abstract

The common marmoset (*Callithrix jacchus*), a nonhuman primate species, is a model organism of great interest due to its translational value in a variety of research settings, including the field of microbiomics. While the composition of the marmoset’s gut microbiome has been somewhat described in captivity, little is known about how gut microbiota interact with each other over time and how they relate to metabolite production. To help answer this, we characterized interactions in the gut microbiome of the common marmoset by calculating the Spearman correlation coefficient between 16S rDNA-derived relative genera abundance data and targeted metabolomics data collected longitudinally from 10 captive marmosets. Association network graphs were used to visualize significant correlations and identify genera and metabolites that have high degree centrality, marking them as more influential within the microbiome. The genus *Clostridium_sensu_stricto_1* engaged in the most metabolomic associations, indicating that it potentially plays a gatekeeping role over metabolites involved in microbial growth and signaling. Its associations with downregulated taurine and bile acids further suggest *Clostridium_sensu_stricto_1* modifies bile acids to exert its influence. *Flavonifractor* and several *Bacteroidales* members had the most bacterial associations and were negatively associated with *Bifidobacterium*, indicating a potential competitive relationship. To further characterize microbiome interactions, we performed hierarchical clustering on significant within-dataset associations and developed a new “Keystone Candidate Score” metric that identified *Clostridium_sensu_stricto_1* and *Alloprevotella* as the most influential bacteria (so-called candidate keystone genera) in the marmoset gut microbiome.

**IMPORTANCE:**

Previous studies have identified significant individuality within the gut microbiome of common marmosets. The reasons for this inter-subject variability and how it relates to health in captivity are poorly understood, owing to a lack of knowledge regarding dynamic interactions between specific microbiota. To that end, this study characterized significant temporal associations between the gut microbiome and metabolome of healthy captive marmosets. Our findings suggest that certain microbial taxa exert a stronger influence within the gut than others. Specifically, *Bifidobacterium* was the most abundant genus and primary driving force behind subject-specific microbiome differences, while Clostridium_sensu_stricto_1 and bacteria from the order Bacteroidales were the main sources, respectively, for significant bacteria-metabolite and bacteria-bacteria associations. Together, this suggests that *Bifidobacterium* may compete with the other taxa for resources and a metabolic niche in the marmoset microbiome.

## INTRODUCTION

The common marmoset is a New World monkey that has become invaluable to researchers due to its fecundity, small size, and complex social behaviors (1, 2). Native to Brazil, it can thrive in environments ranging from tropical forests to semi-arid scrublands due to its omnivorous diet, which gives the marmoset flexibility to choose alternative food sources in times of changing seasonal availability (3, 4). This flexibility is limited, because marmosets are also obligate exudivores that consume tree gum as a large part of their diet (5). As a consequence of their exudivory, functional pathways related to carbohydrate and amino acid metabolism are especially prevalent in the marmoset gut microbiome (6–8). A disparity between wild and captive marmoset diets has been highlighted in the literature (5–7, 9), and diet has been shown to significantly affect the gut microbiome composition and the basal gut metabolome of captive marmosets (7). Despite this, the type/amount of gum to supplement into captive diets, if any, is still debated (5–7, 9), in part due to difficulty defining what a “normal” marmoset microbiome looks like (10).

Unlike the human gut microbiome, which is largely composed of taxa from the phyla of *Bacteroidetes* and *Firmicutes* (11, 12), the gut of wild and captive marmosets can be dominated by any of at least five different phyla (10, 13). This is even true for captive animals that are considered healthy (i.e., lacking a clinical manifestation of disease) (10). The significant individuality observed in studies of marmoset microbiomes has been linked to numerous factors including diet (7), age (14), sex (15), social group membership (13), captivity (6), and importation source (10). Although multiple compositional states of a microbiome can be considered healthy, some may be healthier than others as gastrointestinal diseases are the most reported clinical issue in captive marmosets (16, 17). Particularly relevant to this context, animals from multiple captive facilities have been shown to carry high levels of *Bifidobacterium* spp. (hereafter, bifidobacteria), and several bifidobacteria that are unique to marmosets and other nonhuman primates are shared across facilities (15, 18–26). Furthermore, bifidobacteria have been reported to be even more abundant in the microbiome of wild marmosets compared to captive animals (6, 8). Because some of the bifidobacteria from captive animals can break down complex carbohydrates derived from tree gum (26, 27), high levels of gummivory in wild animals might drive high bifidobacterial abundance. Thus, while many of the bifidobacteria found in marmosets are distinct from those found in humans, the presence and abundance of these organisms could be a general marker of eubiosis for both marmosets and humans (7, 10, 28), and they could play important roles as keystone species.

A keystone bacterial taxon is one that exerts a disproportionate influence on its surrounding environment so that perturbations leading to its removal can permanently alter gut microbiome composition and function (29, 30). Although keystone taxa are typically thought of as being “beneficial” bacteria (i.e., responsible for maintaining a healthy gut by promoting microbiome stability), bacteria that drive the microbiota toward a stable dysbiotic state can also be considered keystone taxa (sometimes differentiated as keystone pathogens) (29, 31). The same host-derived amino acids (AAs) that gut microbes use to synthesize beneficial substances such as short-chain fatty acids (SCFAs) can also be used to produce toxins like ammonia, depending on which species and metabolic pathways are the most active during metabolism of dietary protein (32). Microbial modification of bile acids (BAs) is essential for host cholesterol metabolism but has been implicated in the pathophysiology of multiple diseases (33). Consequently, understanding taxonomic composition and metabolic functions of the microbiome is critical to understanding dynamic host-bacteria, bacteria-diet, and bacteria-bacteria interactions as they may relate to states of health or disease. The common marmoset model provides an excellent opportunity for studying these dynamics given the shared and distinct features of their microbiomes and relatively high rates of gastric disease in captivity, which remain poorly understood. To help meet this need, the goal of the current study is to identify bacteria with a disproportionate influence on the gut metabolome and microbiome (i.e., keystone taxa) in healthy captive marmosets.

The microbiome samples analyzed during this study originated from a previous study that focused on host-microbiome interactions. The original study tested the hypothesis that 6 consecutive days of social isolation-induced stress to the host would cause a significant change in gut microbiome composition compared to control conditions. That study showed no significant effect of social isolation on microbiome composition. Because of the number of samples collected, this enabled us to leverage those samples for additional questions in this current study. (i) How do the microbiome and metabolome change over time in clinically healthy marmosets? (ii) Can central or keystone taxa be identified from network graphical analysis across multiple subjects and what are their functional implications? (iii) Is there any relationship between bacterial abundance and correlation-based degree centrality? The present work aims to address these questions by coanalyzing bacterial and metabolomic abundance data. We hypothesized that *Bifidobacterium* would be a strong candidate keystone genus based on measures of network centrality.

## MATERIALS AND METHODS

### Study design

**Fig 1:**
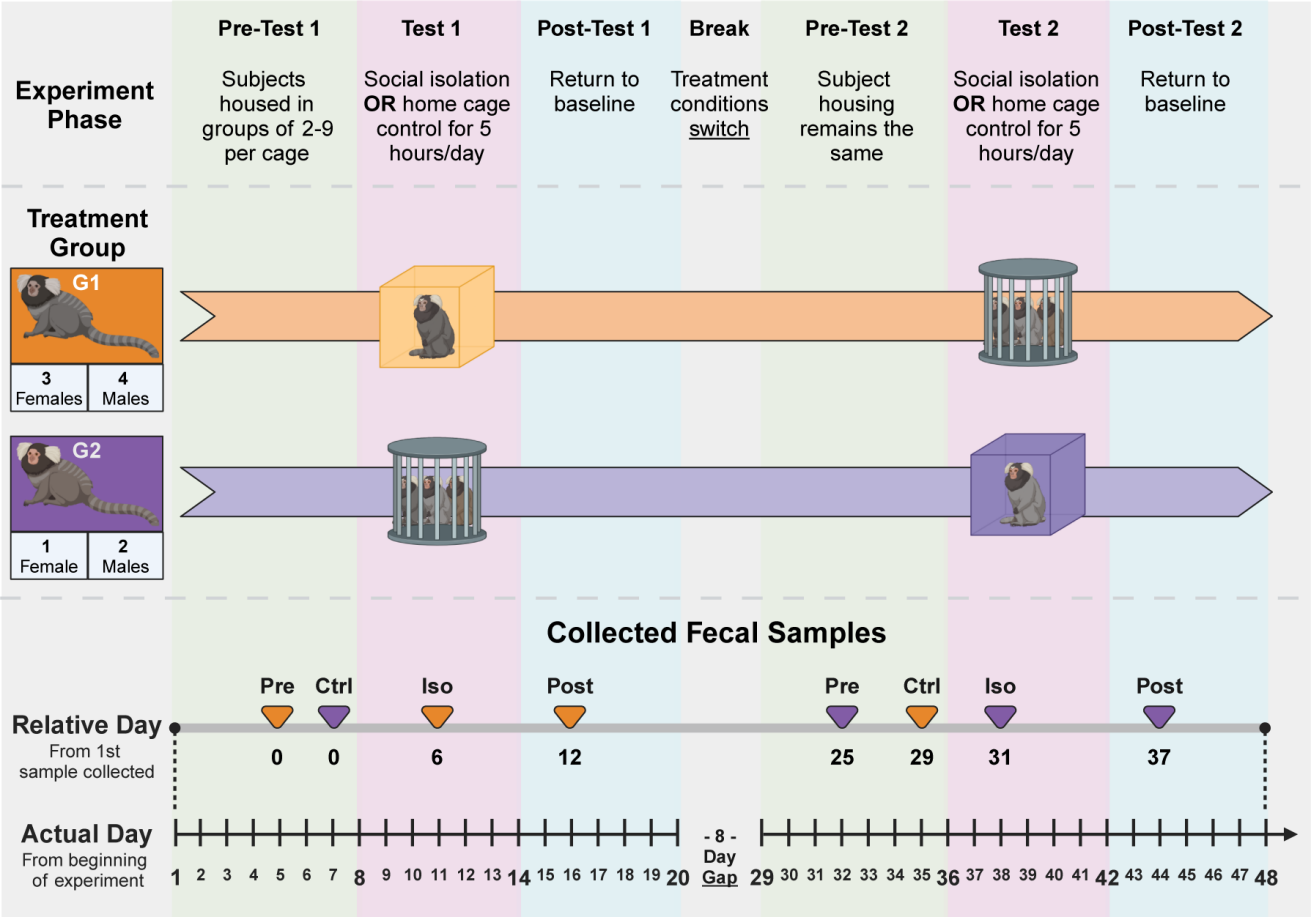
Original study design and sample collection scheme used to generate the samples that were analyzed in the current study. The original study utilized a crossover design that was split into three repeating phases (Experiment Phase) with two treatment groups (Treatment Group). G1 marmosets were subjected to social isolation (marmoset in box), while G2 marmosets remained in their home cage (marmosets behind bars). Treatment conditions were switched halfway through the study (Break). Collected fecal samples (Pre, Iso, Post, and Control) from the original study were reordered in the current study by taking the relative day from the first sample collected. This allowed samples to be compared and analyzed longitudinally, irrespective of treatment groups and treatment conditions from the original study. Figure created in BioRender.

Fecal 16S microbiome and metabolomics data analyzed in the current study were generated using fecal samples collected during a previous unpublished study. In the original study, 10 captive marmosets (aged 1–9 years) were subjected to 5 hours of social isolation (a stressful stimulus for marmosets [34]) for 6 consecutive days. The study utilized a crossover design that split subjects into two treatment groups such that all 10 marmosets served as both the treatment and control subjects (Fig. 1). Three treatment fecal samples and one control fecal sample were collected from each subject (*n* = 40 samples; 4 samples/marmoset × 10 marmosets). Treatment samples were collected approximately 3 days before the isolation challenge (Pre-Isolation or Pre), 3 days into the challenge (Iso), and 3 days after the end of the challenge (Post-Isolation or Post). A control sample (Ctrl or Control) was collected from each subject either 3 weeks after or 4 weeks before isolation, depending on the animal’s treatment group membership. Collected fecal samples were aliquoted and frozen before being stored at −80°C. Further details about the original study, including subject diet, housing conditions, and social isolation, can be found in the Supplementary Methods.

When samples were collected, the date of collection was recorded for each sample. This allowed longitudinal analysis to be conducted by ordering each within-subject replicate chronologically and counting the number of days from the first time point, which was labeled relative day 0 (Fig. 1; Fig. S1). In other words, the first sample collected from each subject was used as an anchor point, and all subsequent samples were ordered with respect to that anchor point.

### 16S rDNA microbiome data generation and analysis

DNA extraction using the BioSprint 96 One-For-All Vet Kit (Indical Biosciences) was performed at the Nebraska Food for Health Center (Lincoln, NE) using manufacturer’s instructions. DNA quality and concentration were evaluated using Qubit fluorometer v4.0 (Invitrogen Corporation). Using the extracted DNA, the V4 region of the 16S rRNA gene was amplified using a previously described protocol and primers (15). Sequencing was performed on the Illumina MiSeq platform (35).

Creation and denoising of an amplicon sequence variant (ASV) feature table, as well as taxonomy classification, filtering, and relative abundance calculation, were performed using various plugins within the QIIME 2 package (36). Removal of primer sequences was performed with the Cutadapt plugin (37), while ASV table creation and denoising were performed with the DADA2 plugin (38). Taxonomy classification was performed with a QIIME 2 naive Bayes classifier trained on full 16S rDNA sequences extracted from the Silva database (138 SSU Ref NR 99). Sequences were extracted using the QIIME 2 “extract_reads” function if they had a forward primer sequence of “GTGCCAGCMGCCGCGGTAA” or a reverse primer sequence of “GGACTACHVGGGTWTCTAAT.” After taxonomy classification, features were removed if they were only present in a single sample or were classified as mitochondria or chloroplasts. Samples with <1,000 total reads (*n* = 2) were removed. After filtering, microbial taxa were collapsed to the genus level (39), and the relative abundance of the resultant 54 genera was calculated for each sample.

### Metabolomic data generation and analysis

Metabolomics data comprised three targeted panels: An SCFA panel with five metabolites, a BA panel with 16 metabolites, and an AA panel with 26 metabolites. The SCFA and BA panels were chosen because gut microbiota play a known role in their production, providing definitive evidence for microbial involvement if differential abundance is observed. The AA panel was chosen because the marmoset microbiome plays a prominent role in AA metabolism (6, 7) and because AAs are precursors for many neurotransmitters (e.g., γ-aminobutyric acid or GABA). Targeted analysis of SCFAs and BAs was conducted using GC-MS and LC-MS/MS, respectively. Sample extraction and metabolite quantitation for these steps were carried out as previously described (40) and described in the Supplementary Methods. Targeted analysis of AAs was conducted using HPLC. AAs were extracted from the samples using a liquid-liquid extraction, as described previously in 41, after which the dried samples were resuspended in 20 mM HCl and an aliquot used for derivatization using AccQ-Tag chemistry (Waters, Milford, MA, USA), as per the manufacturer’s protocol. The derivatized compounds were then analyzed using the Agilent UHPLC Infinity II using a UV detector. For LC separation, an ACCQ-TAG ULTRA C18 1.7 um (2.1 × 100 mm, Waters) was used at a flow rate of 0.7 mL/min at 48°C with a quaternary gradient. The gradient of the mobile phases was as follows: 10% A (100% Eluent A), 0% B (10% Eluent B), 90% C (100% H_2_O), 0% D (100% Eluent B) from 0 to 0.29 min; to 9.1% A, 70% B, 20.9% C, 0% D in 4.55 min; to 8% A, 15.6% B, 58.9% C, 17.5% D in 1.61 min; to 7.8% A, 0% B, 71.9% C, 20.3% D in 0.39 min; to 13.7% A, 0% B, 36.3% C, 50% D in 0.6 min; hold for 1.25 min; back to initial conditions in 0.09 min. The absorbance was measured at 260 nm. For quantification, an external standard curve was prepared using a series of standard samples containing different concentrations of AAs.

Metabolites from all three targeted panels were concatenated together and processed in a single matrix with samples as rows and metabolites as columns. All metabolite abundances were converted to the unit of µg/g before continuing. Metabolite InChIKeys were cross-checked against the PubChem database with the PUG REST API. Metabolites (features) that did not have a corresponding match or that had more than 70% missing values were removed (42). Remaining missing values were imputed with the feature-wise median, and features were median-scaled (divided by the feature-wise median excluding imputed values) so that each metabolite had a median of 1. Median-scaled metabolites were used for all subsequent analysis steps. Metabolomics data were normalized by panel (i.e., divided by the mean abundance of all metabolites within a panel on a per-sample basis) before performing any ordination-based analyses (e.g., PERMANOVA and principal coordinates analysis). A subset of metabolome samples (*n* = 2) was excluded from correlation and network analyses because their corresponding microbiome samples had low read abundance and were removed.

### Statistical analyses

All statistical analyses were based on the analytical workflow and code provided by Lloyd-Price et al. (43). Unless otherwise specified, all software analyses were performed using a Jupyter Lab notebook within a custom Conda environment that was hosted on the University of Nebraska’s Holland Computing Center.

### Principal coordinates analysis

Metabolomics data were normalized by panel, and relative genera abundance data were row-sum normalized before conducting principal coordinates analysis (PCoA). Sample-wise Bray-Curtis dissimilarity was calculated for microbiome and metabolomics data using the “dsvdis” function in the labdsv R package (44). PCoA plots were generated and visualized with the vegan and ggplot2 packages (45, 46), respectively.

### Omnibus testing

Omnibus testing was performed on normalized Bray-Curtis dissimilarity matrices for metabolite and relative genera abundance with the R package vegan. Microbiome abundance samples with <1,000 total reads were retained because PERMANOVA requires a balanced number of samples for permuting within the subject group. Inter- and intra-subject variance with marginal effects was assessed with PERMANOVA (9,999 permutations). Variance explained by the model “Dissimilarity Matrix ~ Treatment_Condition + Relative_Day” was calculated by permuting within each subject and treatment group. Variance explained by the model “Dissimilarity Matrix ~ Sex + Treatment_Group + Age” was calculated by permuting freely between subjects.

### Differential feature analysis

Differential feature analysis was performed to find features that varied as a result of treatment conditions from the original study (Pre, Stress, Post, and Control). Differential alpha and beta diversity testing were performed with QIIME 2 after rarefying to a sequencing depth of 1,937 reads. Tests performed included pairwise Kruskal-Wallis and PERMANOVA to test for differences in alpha and beta diversity, respectively. Differential genera abundance was tested with linear discriminant analysis with Effect Size measurements (LEfSe) at the genus level using default parameters. Differential metabolite abundance was assessed by fitting median-scaled, log_2_ +1-transformed metabolomics data to a linear mixed-effects model using the following formula with subject (Individual) as a random effect:

Feature ~ Treatment_Condition + Individual

All p-values were adjusted with the Benjamini-Hochberg procedure and considered significant at a false discovery rate (FDR) of 5%.

### Correlation analysis

Data sets were transformed to increase the linearity and reduce the influence of outliers. Metabolomics data were log2-transformed with a pseudocount of 1, and microbiome data were arcsine square-root-transformed (43). A feature-wise linear model was fitted to the data with subject as the independent fixed effect. The Pearson residuals were extracted from the fitted model in order to remove any variance associated with the independent variable so that any remaining variance should be due to time (43, 47). Correlation analysis between feature residuals was performed using Hierarchical All-against-All association (HAllA) testing v0.8.20 (48). Spearman correlation was used as a similarity metric, and AllA (a variant of HAllA that performs all-against-all association testing without hierarchically clustering features beforehand) was used for within-dataset association testing. Because within-dataset association matrices were symmetrical, only the upper triangle of within-dataset association matrices was used for further analysis. Associations were considered significant if they had a Benjamini-Hochberg FDR-adjusted p-value (q-value) <0.05. Heatmaps were created using HAllA, which creates a heatmap for each association matrix by default.

### Eigendecomposition, A/B classification, and hierarchical clustering

Eigendecomposition was performed on within-dataset Spearman correlation matrices with the Python package scikit-learn using the PCA function. The first principal component (eigenvector) was extracted from the eigendecomposed matrix and used to classify each feature into type A (positive-valued eigenvector) or type B (negative-valued eigenvector). Hierarchical clustering was performed on within-dataset Spearman correlation matrices with seaborn’s clustermap function, with “correlation” specified as the metric parameter.

### Network graph construction and inference

Significant associations were exported into Cytoscape to create an association network. All network visualizations were created with Cytoscape with the Perfuse Force Directed Layout option. Network measures (e.g., degree, betweenness centrality, and network density) were calculated with the Cytoscape network analyzer. Network graph clustering was performed using MCODE with default settings. Slight modifications to the networks and legends created in Cytoscape, including increasing text size, replacing legend text, reducing distance between network clusters, and shortening of peripheral edges to increase readability, were performed in Adobe Illustrator.

### Network graph degree distribution and fitting of power-law distribution

Network graphs of metabolite-metabolite, bacteria-metabolite, and bacteria-bacteria associations were exported from Cytoscape in CSV format, and their degree distributions were calculated and plotted in R. For each degree distribution, a power-law distribution was fitted, and goodness of fit was assessed with the poweRlaw package (49). This package was also used to calculate the significance of the fit by performing a Kolmogorov-Smirnov test using 5,000 bootstrap iterations and to compare the fit of the power-law distribution to those of the log-normal, exponential, and Poisson distributions using Vuong’s method (50). Output of these tests, as well as a brief description for their interpretation, can be found in File S1. Steps to increase the readability of generated figures, including relabeling of axes, changing line colors and text size, and extracting graphs of interest from multipanel graphs, were performed in Adobe Illustrator.

## RESULTS

### Differentially abundant gut metabolites are the product of time instead of treatment condition

Although multiple samples were collected from each marmoset, they were taken at slightly different time points in each animal (Fig. 1; Fig S1). Together with the crossover design of the original study, this precluded the use of a conventional sample ordering scheme due to a conflation of time point number with treatment condition. Accordingly, significance testing was first performed to determine if testing conditions from the original study (Pre, Iso, Post, and Control) had a substantial effect on the microbiome or metabolome.

PERMANOVA testing for inter-subject marginal effects of age, treatment group, and sex was performed on normalized Bray-Curtis dissimilarity matrices for microbiome and metabolite abundance and detected no significant results. Statistical analyses for differential microbiome feature abundance as a product of treatment condition detected no significant changes. Intra-subject PERMANOVA yielded a significant difference in metabolite abundance from treatment condition, but only with relative day included in the model. Subsequent fitting of metabolomics data to a mixed-effects model detected two differentially abundant (DA) metabolites in Iso samples, 10 DA metabolites in Pre samples, and no DA metabolites in Post samples, compared to controls (Table 1). The fact that most significant changes in metabolite abundance were detected in Pre vs Control samples (which were collected on different days under otherwise identical experimental conditions), and that no significant metabolites were unique to the Iso or Post conditions, strongly suggests that the observed changes in abundance were instead a product of time. Due to this, all further analyses focused on characterizing the longitudinal variation.

**TABLE 1:** Differentially abundant metabolites by treatment condition (FDR-adjusted q-value <0.05)^*a*^

Metabolite name	Treatment condition	Log2FC	p-value	q-value
alpha-Muricholic acid	Pre	−0.718	0.003	0.031
Cholic acid	Pre	−1.800	0.012	0.046
Glycocholic acid	Pre	−1.119	0.001	0.023
Glycocholic acid	Iso	−1.006	0.002	0.032
Glycochenodeoxycholic acid	Pre	−0.795	0.001	0.024
Glycodeoxycholic acid	Pre	−0.557	0.008	0.035
Glycodeoxycholic acid	Iso	−0.731	0.001	0.031
Taurocholic acid	Pre	−2.691	0.004	0.031
Taurodeoxycholic acid	Pre	−2.005	0.008	0.031
Taurine	Pre	−1.968	0.006	0.031
Threonine	Pre	0.296	0.004	0.031
Lysine	Pre	0.447	0.008	0.035

^*a*^ Metabolites are identified by their common name, as well as the treatment condition(s) in which they were differentially abundant, and the log2 fold change compared to the control group.

### Statistical analysis of within-dataset Pearson residuals shows significant correlations among metabolomics and microbiomics data

To separate the longitudinal variation of the microbiome and metabolome from non-longitudinal variation, a linear regression model of subject-specific effects was fitted to each data set. Pearson residuals were extracted from the fitted model to minimize non-temporal variance between features, and the Spearman correlation coefficient between extracted residuals was calculated with HAllA (48). After FDR adjustment, 275 significant metabolite-metabolite correlations and 74 significant bacteria-bacteria correlations were detected (Fig. S2A and B).

To explore the disparity in the number of significant correlations among microbiomics compared to metabolomics data, PCoA was performed for both data types. Although a slight temporal gradient could be seen, there was no obvious separation between any of the metadata variables measured during the study (Fig. S3A and B). The temporal gradient was more pronounced in the metabolomics data, with a corresponding increase in variance captured by the first principal coordinate compared to microbiome data, suggesting that metabolites underwent more fluctuations over time.

### Metabolite and bacteria correlation matrices show distinct clusters after hierarchical clustering and eigendecomposition-based A/B labeling

**Fig 2:**
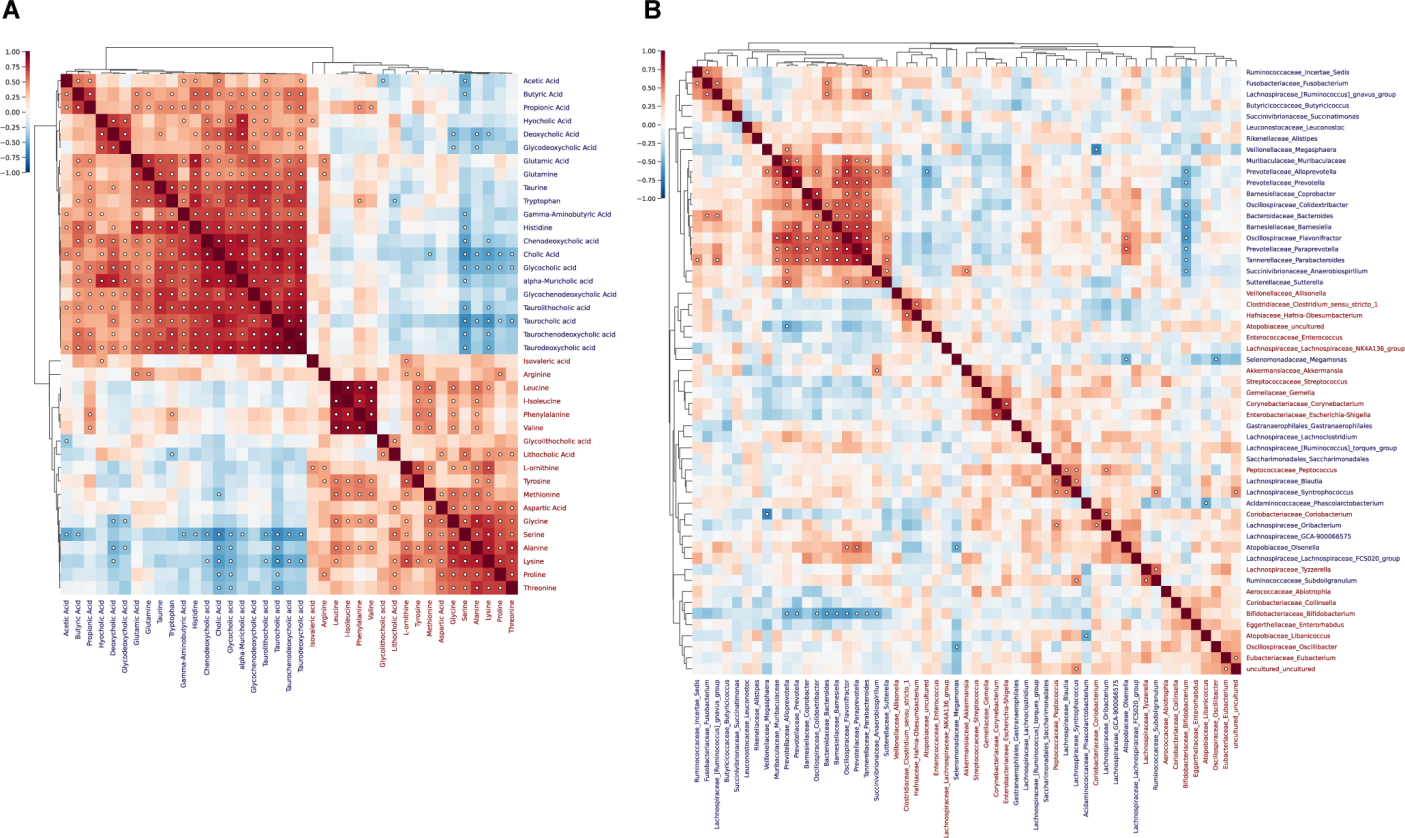
Hierarchical clustering and eigendecomposition reveal the existence of substructures within metabolite-metabolite and bacteria-bacteria correlation matrices. Clustermaps depicting within-dataset Spearman correlation coefficient and hierarchical clustering dendrograms for (**A**) metabolomics and (**B**) relative genera abundance profiles. White dots depict associations with marginal FDR-adjusted *P*-value < 0.05. Features were classified as type A or type B features according to the sign of their first principal component (eigenvector). Red text labels correspond to features with positive-valued eigenvectors (type A), while blue labels correspond to features with negative-valued eigenvectors (type B).

To further clarify relationships between features in the within-dataset correlation matrices, hierarchical clustering and eigendecomposition were performed (Fig. 2A and B). Features were classified as type A or type B features according to the sign of their eigenvector (51), with positive-valued and negative-valued eigenvectors corresponding to types A and B, respectively. Upon inspection, features with the same classification tended to correlate with each other and cluster together, while features with opposite classifications tended to be anti-correlated (Fig. 2A). Moreover, A/B membership and hierarchical clustering reflected biological relationships between features. Aliphatic, hydroxylic, positively charged, and branched-chain AAs were classified as type A, and all but 2 BAs and 1 SCFA were classified as type B. Remaining polar, charged, and aromatic AAs were classified between types A and B, suggesting that these classes of AAs behave more erratically.

The relationship between positive/negative correlation and A/B membership was also present (though less pronounced) in the bacteria-bacteria correlation matrix as almost all significant negative correlations were between microbes of opposite labels. Indeed, only 14 of the 74 significant bacteria-bacteria correlations were negative (Fig. 2B), with 9 of those involving type A *Bifidobacterium* and two others involving type B *Megamonas*. Phylogenetically, the phyla *Fusobacteriota*, *Patescibacteria*, and *Bacteroidota* were exclusively classified as type B, *Verrucomicrobiota* and all but one *Actinobacteriota* were classified as type A, while *Firmicutes* and *Proteobacteria* were split more evenly between types A and B (Fig. S4). Together, these results suggest that chemically similar metabolites, and phylogenetically similar bacteria to a lesser extent, preferentially correlate with each other over time. Intrigued by this notion, we next examined bacteria-metabolite relationships.

### Combined data analysis provides interpretable biological relationships between bacteria and metabolites

**Fig 3:**
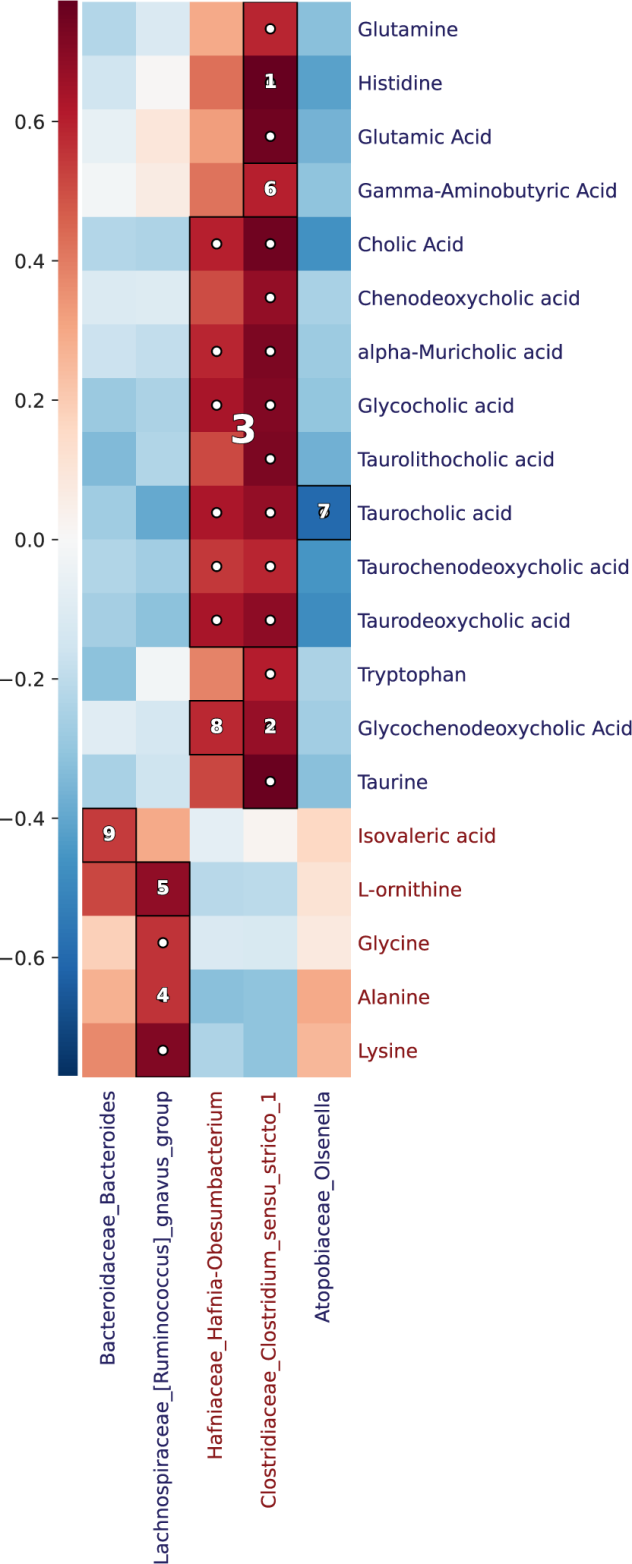
*Clostridium_sensu_stricto_1* is correlated with multiple taurobile acids and taurine, suggesting it modifies bile acids by conjugating/deconjugating taurine. Heatmap depicting the Spearman correlation coefficient between metabolomics and relative genera. White dots depict associations with marginal FDR-adjusted *P*-value < 0.05. Numbers are clusters ranked by increasing significance, as defined by HAllA. Red and blue labels correspond, respectively, to features classified as type A and type B in either bacteria-bacteria or metabolite-metabolite correlation matrices.

The bacteria-metabolite Spearman correlation coefficient was calculated for Pearson residuals of the previously described fitted linear models. After FDR adjustment, 28 significant bacteria-metabolite correlations were detected (Fig. 3). Intriguingly, after assigning within-dataset A/B labels to those correlations, five correlations involved type A metabolites, six involved type B microbes, and 5 of those six were between type A metabolites and type B microbes (Fig. 3). This, despite 40% of microbes and metabolites being classified as type A, indicates a preferential correlation between type A microbes and type B metabolites.

In addition to calculating the significance of bacteria-metabolite correlations, HAllA was used to identify and rank clusters in the correlation matrix. A correlation of *Clostridium_sensu_stricto_1* with glutamine, glutamate, and histidine received the highest ranking, while a correlation between this genus and GABA received a ranking of 6. *Clostridium_sensu_stricto_1* was also in the second- and third-highest-ranked HAllA clusters, which, respectively, included taurine and multiple tauroBAs, a strong indication that *Clostridium_sensu_stricto_1* spp. could participate in the conjugation/deconjugation of taurine-derived BAs (52). Because circulating taurine levels are higher in marmosets compared to humans (53), microorganisms possessing control over bacterial taurine reserves would be well-positioned to act as gatekeepers that modulate downstream flora (54, 55). *Clostridium_sensu_stricto_1* was particularly notable in this regard, participating in over half of all significant bacteria-metabolite correlations (including 7/10 metabolites that were DA between experiment phases). Prompted by this, within- and between-dataset relationships were investigated simultaneously with the use of the graphical analysis methodology.

### Bacterial association network graphs display a power-law distribution

**Fig 4:**
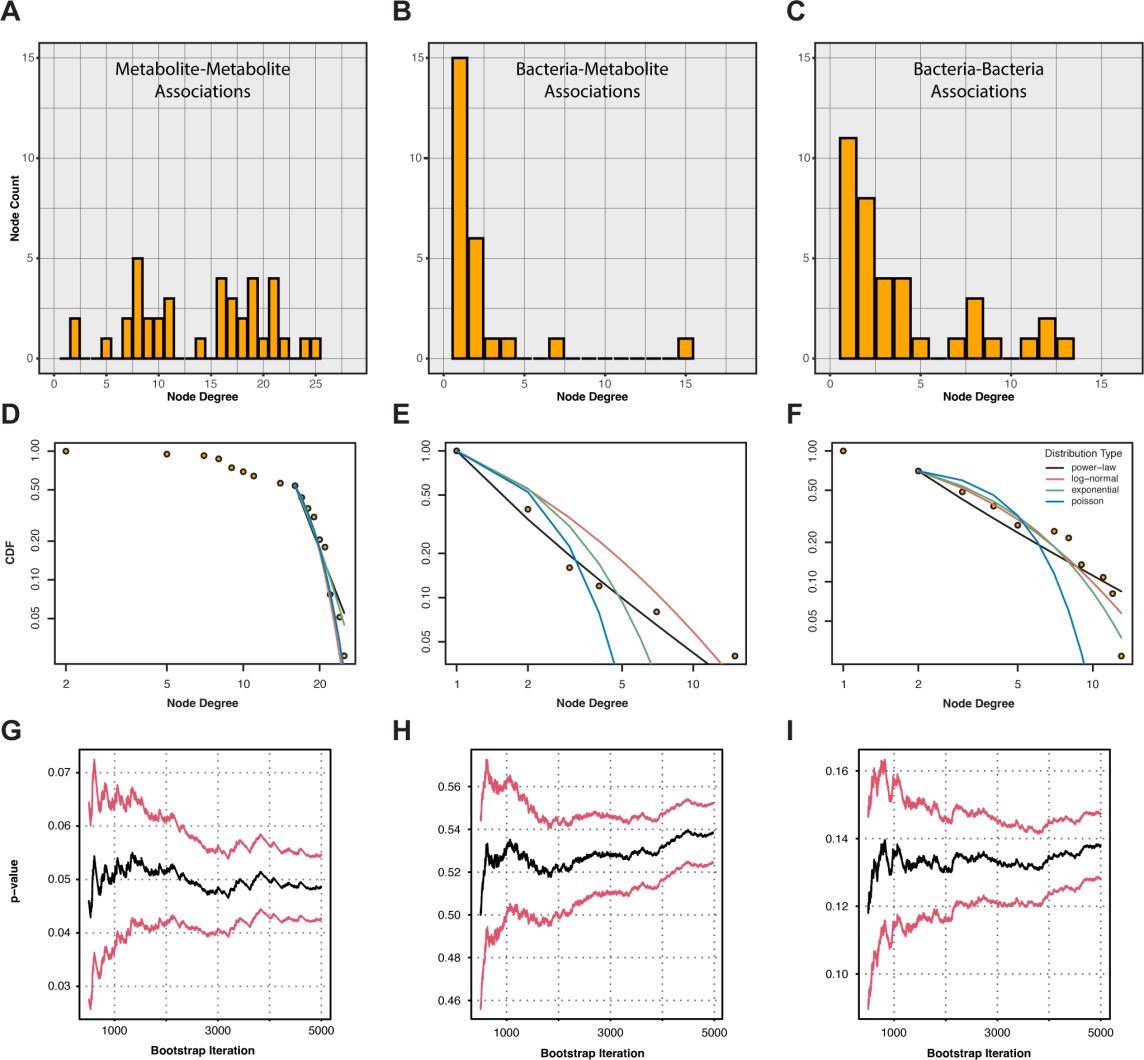
Bacteria-metabolite and bacteria-bacteria degree distributions approximate a power-law distribution, making them scale-free. (**A through C**) Frequency histogram depicting node degree distributions for (**A**) metabolite metabolite; (**B**) bacteria-metabolite; and (**C**) bacteria-bacteria association network graphs. (**D through F**) Cumulative distribution functions (CDFs) for (**D**) metabolite-metabolite; (**E**) bacteria-metabolite; and (**F**) bacteria-bacteria degree distributions fitted to several heavy-tailed distributions, including a power-law distribution. Cumulative probabilities of each x-axis input to a given CDF are depicted along the y-axis on a log scale. Each colored line represents the maximum likelihood fit to the data from a different distribution. Lines for all distributions are colored consistently according to the key in (**F**). (**G through I**) Plausibility that the CDFs for (**G**) metabolite-metabolite; (**H**) bacteria metabolite; and (**I**) bacteria-bacteria degree distributions were from a power-law distribution was assessed with a p-value that was calculated from a bootstrap procedure, implemented in R using the poweRlaw package. Significance (*P* < 0.05) indicates low plausibility, as *P* tests the null hypothesis that the data are from a power-law distribution. Black lines denote the cumulative mean of *P* across 5,000 bootstrap iterations, while red lines denote the 95% confidence interval.

A network graph was constructed from significant bacteria-bacteria, bacteria-metabolite, and metabolite-metabolite correlations to identify microbes and metabolites with a high number of correlations (associations) across data sets. The network graph was partitioned into three separate subgraphs corresponding to the three fundamental types of associations listed above. Plotting the degree distributions for the three subgraphs revealed that bacterial association networks might follow a power-law distribution (Fig. 4A through C), which would make them scale-free, with the likely presence of hub nodes that could be interpreted as keystone taxa (29). Formal testing of this idea with the poweRlaw package (49) showed that the metabolite-metabolite subgraph fitted poorly to a power-law distribution, fitting significantly better to the exponential distribution instead (Fig. 4D and G; Supplementary Results). The bacteria-metabolite and bacteria-bacteria subgraphs fitted well to a power-law distribution, demonstrating scale-free topology (Fig. 4E, F, H and I; Supplementary Results).

### Graphical analysis with network partitioning identifies central metabolites and bacteria, including a cluster of *Bacteroidales*

**Fig 5:**
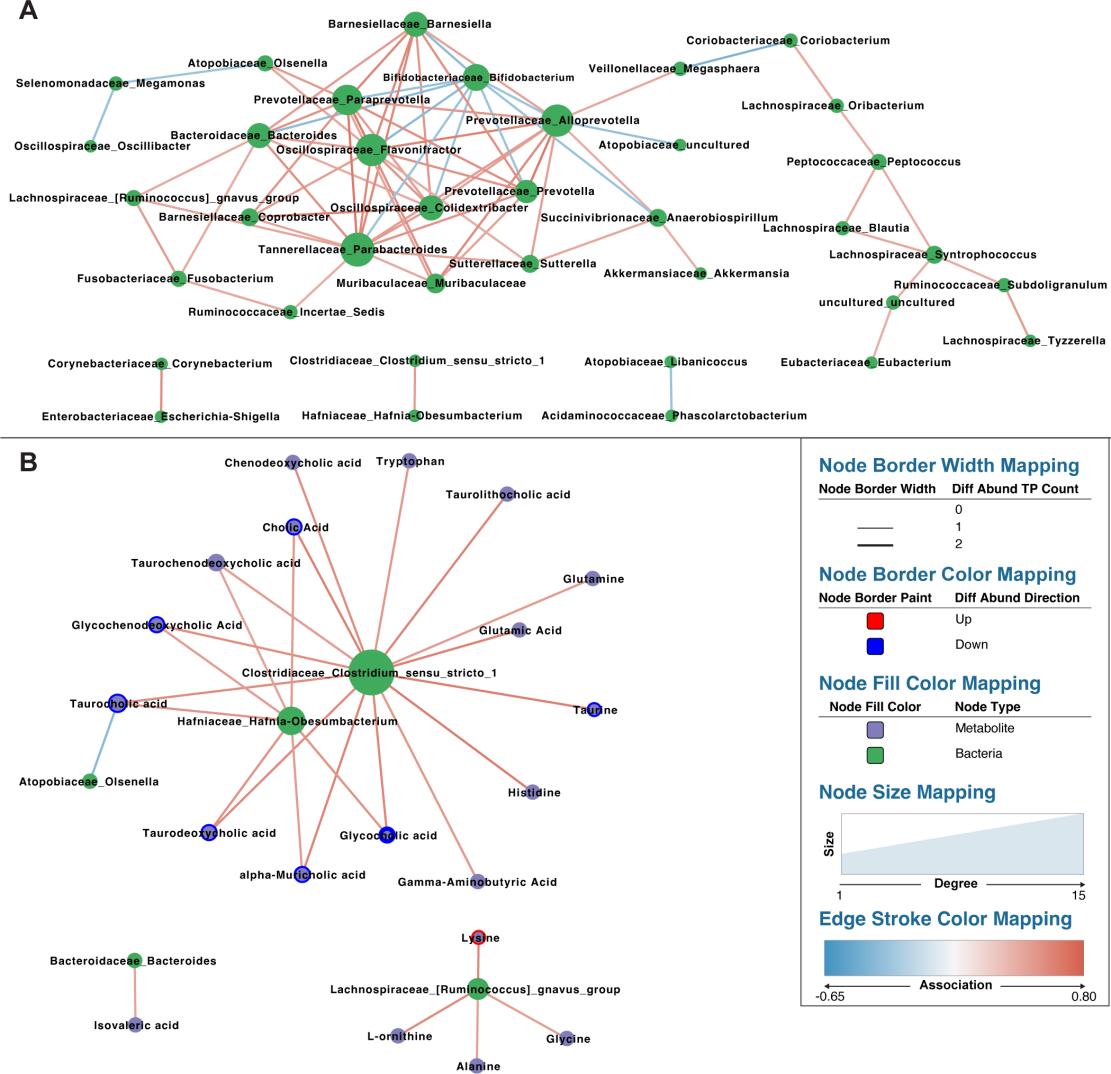
*Clostridium_sensu_stricto_1*’s correlation with downregulated taurocholic acid connects it to the bacteria-bacteria association graph via *Olsenella*, indicating it could play a regulatory role in the microbiome. Association networks for correlation matrices generated by HAllA with a q-value threshold of 0.05. (**A**) Bacteria-bacteria and (**B**) bacteria-metabolite correlations for all metabolite panels combined. Green nodes are bacterial genera, and purple nodes are metabolites. Node size corresponds to degree, while edge color corresponds to the sign and strength of associations. Differentially abundant metabolites are denoted by colored node borders, with the thickness of each border denoting the number of experimental phases in which the abundance change was significant.

Closer inspection of the bacterial association subgraphs revealed nodes that were more central than others (i.e., possessing higher degree centrality). The most central node in the bacteria-bacteria subgraph was *Parabacteroides* with a degree of 13, followed by *Flavonifractor* and *Alloprevotella* with a degree of 12 (Fig. 5A). The most central nodes in the bacteria-metabolite subgraph were *Clostridium_sensu_stricto_1* and *Hafnia-Obesumbacterium* with respective degrees of 15 and 7 (Fig. 5B). The most central metabolite in this subgraph was the BA taurocholic acid with a degree of 3.

Bacterial association subgraphs were scale-free and more topologically diverse than the metabolite-metabolite subgraphs, which were largely homogenous in their degree distribution (Fig. 4; Table S2). Betweenness centrality, which measures the proportion of shortest paths through the network that intersect a given node, was therefore calculated for the largest connected component of the bacteria-bacteria and bacteria-metabolite subgraphs. *Alloprevotella* and *Megasphaera* had the highest betweenness centrality in the bacteria-bacteria subgraph (0.54 and 0.43, respectively). *Clostridium_sensu_stricto_1* and taurocholic acid had the highest betweenness centrality in the bacteria-metabolite subgraph (0.83 and 0.13, respectively).

Graphical analysis also identified small groups of highly interconnected nodes resembling clusters. To confirm this notion, the Cytoscape plugin MCODE was used to find clusters from densely connected regions in the bacteria-metabolite and bacteria-bacteria subgraphs (56). MCODE found two clusters within the bacteria-bacteria subgraph and 0 within the bacteria-metabolite subgraph (Fig. S5). The first bacteria-bacteria cluster (henceforth referred to as the *Bacteroidales* cluster) consisted of *Bifidobacterium* along with 2 *Oscillospiraceae* family members and 7 *Bacteroidales* order members. The second cluster consisted of *Clostridia* members *Peptococcus*, *Blautia*, and *Syntrophococcus*.

### Network analysis identifies *Clostridium_sensu_stricto_*1 and *Alloprevotella* as the strongest candidate keystone genera in the marmoset microbiome

In order to reach a meaningful conclusion from the various network measures utilized in this study, and because the bacterial association networks had scale-free topology, we sought to identify keystone taxa from the putative interactions across the bacterial association subgraphs. Microbial co-occurrence networks such as the one in this study can be used to identify putative keystone taxa by interpreting central nodes as being more influential with the stipulation that further evidence (e.g., structural equation modeling and experimental manipulation) is required to confidently label keystones (29, 57). To accommodate this limitation, candidate keystone genera were identified and ranked by aggregating the MCODE cluster status, number of DA metabolite associations, degree centrality, and betweenness centrality of each node in the bacteria-bacteria and bacteria-metabolite subgraphs into a single composite “Keystone Candidate Score” (KCS; Table 2). MCODE identifies clusters from locally dense seed nodes (56), and high degree and closeness centrality are considered the best indicators of keystoneness inferred from co-occurrence networks (57). While betweenness centrality is also considered an indicator of keystoneness, there is conflicting consensus in the literature on whether high or low betweenness centrality is a better indicator of keystoneness (29). Finally, we reasoned that keystone taxa would be directly or indirectly responsible for significant changes in the metabolite abundance over time, and so bacteria-metabolite associations with DA metabolites were given higher preference in our scoring. The strongest keystone candidates would therefore be MCODE seed nodes with many DA metabolite associations, high degree centrality, and high/low betweenness centrality across both the bacteria-bacteria and bacteria-metabolite subgraphs.

**TABLE 2:** Top 20 microbial genera ranked by keystone candidate score^*a*^

Genus	KCS	MCODE status	Diff. Metab	Degree centrality		HiLo betweenness centrality	
				Bact-Bact	Bact-Metab	Bact-Bact	Bact-Metab
*Clostridium_sensu_stricto_1*	26	0	7	1	15	0	3
*Alloprevotella*	20	5	0	12	0	3	0
*Parabacteroides*	18	5	0	13	0	0	0
*Flavonifractor*	17	5	0	12	0	0	0
*Prevotella*	17	10	0	7	0	0	0
*Hafnia-Obesumbacterium*	17	0	6	1	7	0	3
*Paraprevotella*	16	5	0	11	0	0	0
*Blautia*	15	10	0	2	0	3	0
*Bifidobacterium*	14	5	0	9	0	0	0
*Bacteroides*	14	5	0	8	1	0	0
*Barnesiella*	13	5	0	8	0	0	0
*Colidextribacter*	13	5	0	8	0	0	0
*Muribaculaceae*	13	5	0	5	0	3	0
*Syntrophococcus*	9	5	0	4	0	0	0
*Peptococcus*	8	5	0	3	0	0	0
*Olsenella*	8	0	1	3	1	0	3
[Ruminococcus]_gnavus _group	7	0	0	3	4	0	0
*Coprobacter*	7	0	0	4	0	3	0
*Megasphaera*	5	0	0	2	0	3	0
*Coriobacterium*	5	0	0	2	0	3	0

^*a*^ Keystone candidate score (KCS) is aggregated from each taxon’s MCODE cluster status, number of correlations with differentially abundant metabolites (Diff. Metab), and degree and betweenness centrality on the bacteria-bacteria (Bact-Bact) and bacteria-metabolite (Bact-Metab) subgraphs.

Accordingly, the KCS was calculated for each node by aggregating across six subcomponent scores: (i) MCODE status, a ternary score of 0 for nodes that were not members of an MCODE cluster, five for nodes that were cluster members (excluding seed nodes), and 10 for nodes that were seed nodes within a cluster; (ii) differential metabolites, the number of DA metabolites associated with a given bacterium; (iii to iv) the node’s degree centrality for bacteria-bacteria and bacteria-metabolite subgraphs; (v to vi) HiLo betweenness centrality, a binary score of 3 for nodes with the three highest/lowest betweenness centralities from the largest connected component of the bacteria-bacteria and bacteria-metabolite subgraphs, or 0 for remaining nodes. In the event of a tie for the top three highest/lowest betweenness centralities, the node with the highest degree centrality was selected. Subsequent ties were broken by the highest closeness centrality. After performing the calculations, *Clostridium_sensu_stricto_1* had the highest KCS of 26, followed by *Alloprevotella* with a KCS of 20 and *Parabacteroides* with a KCS of 18.

### *Bifidobacterium* abundance corresponds well to beta diversity between microbiome samples

**Fig 6:**
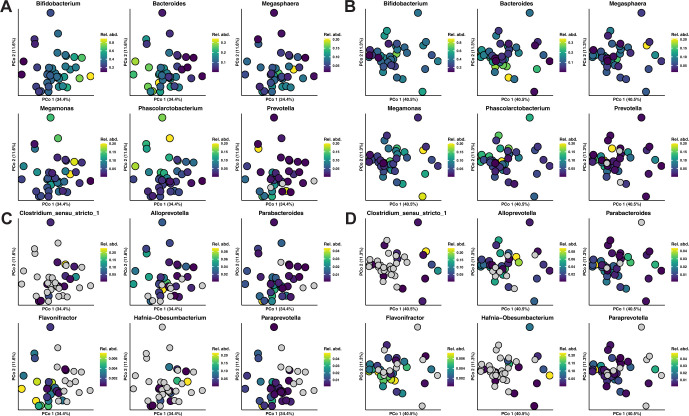
Changes in *Bifidobacterium* abundance explain beta diversity between 16S rDNA samples better than that of other highly abundant or highly central taxa. (**A, C**) Principal coordinates plots of Bray-Curtis dissimilarity for 16S rDNA sample profiles, colored by the relative abundance of the six most abundant and the six most central genera, respectively, in descending order from left to right and top to bottom. (**B, D**) Principal coordinates plots of Bray-Curtis dissimilarity for metabolome. Sample profiles colored by the same genera as those in A and C. Gray dots correspond to samples where no reads were detected for a given genus. Two samples (Marm_02 Relative_Day 39 and Marm_07 Relative_Day 11) were excluded due to low total read abundance.

Because our proposed KCS metric is primarily derived from measures of network centrality and does not take bacterial abundance into account, we next explored how network centrality (and KCS by proxy) affected beta diversity compared to the overall abundance. Accordingly, a PCoA plot of microbiome Bray-Curtis dissimilarity was created. Each sample in the plot was annotated by relative genera abundance, starting with the six most abundant taxa (Fig. 6A) followed by the six highest-ranked taxa by KCS (excluding *Prevotella* due to its inclusion in the previous taxa list; Fig. 6C). Plotting revealed a clear gradient of *Bifidobacterium* abundance along the first principal coordinate, indicating that it was the primary source of 34.4% explained variance. Less pronounced gradients were also present for *Parabacteroides*, *Flavonifractor,* and *Paraprevotella*, indicating that even small shifts in these central taxa have a noticeable impact on beta diversity between samples. Similar plotting of metabolome samples (Fig. 6B and D) revealed that *Clostridium_sensu_stricto_1* and *Hafnia-Obesumbacterium* absence/abundance contributed to metabolite variation between samples, indicating that these bacteria are influential within the gut metabolome despite their low overall abundance. *Alloprevotella* abundance exhibited no discernible trend in either plot however, suggesting that its high bacteria-bacteria network betweenness centrality did not translate to greater influence.

## DISCUSSION

Previous studies have shown that bifidobacteria are important residents of the marmoset gut (6, 8, 26), which can vary in abundance as a product of multiple environmental factors including diet (7) and captivity status (6). In this study, we characterized significant temporal associations between the gut microbiome and metabolome of healthy captive marmosets using clustering and graphical analysis methods. After demonstrating that bacterial association networks follow a scale-free topology, we used the KCS, a novel aggregate scoring metric for network graphs, to test the hypothesis that *Bifidobacterium* would be a strong candidate keystone genus based on measures of network centrality.

*Bifidobacterium* was the dominant genus for all but one subject in this study (Marm_01; Fig. S6), contrasting with the results of some captive studies (6, 10, 17, 58) and agreeing with others (24, 26, 40). In light of its abundance, which was a clear driver of beta diversity between samples (Fig. 6A), it is surprising that *Bifidobacterium* only ranked number 10 in terms of KCS, defying our hypothesis. The fact that it tied with the second-most-abundant genus, *Bacteroides*, is also interesting and suggests that taxa with high average abundance typically have a low corresponding KCS (*Prevotella* being one possible exception). The inverse of this may also be true as it is not unusual for low-abundance organisms to be keystones. Several proposed human commensal keystones have been reported at <30% sample prevalence and <1% mean relative abundance (59, 60), comparable to the high-KCS genera identified here. There is no relationship between a taxon’s keystoneness and its abundance *per se* (29), but given the challenge of differentiating between highly abundant keystone taxa and those that are influential simply by virtue of their high biomass (so-called foundation taxa), it is unsurprising that keystone taxa are often restricted to be uncommon (61). One could argue that *Bifidobacterium* is a strong keystone candidate due to evidence that it is essential for promoting a healthy marmoset gut microbiome (8). The fact that it was anticorrelated with the other nodes in the *Bacteroidales* MCODE cluster could also suggest that it plays a modulatory role in the microbiome, which fits with the canonical understanding of a keystone taxon’s role. A counterargument can be made, however, that its high abundance and susceptibility to changing diet make *Bifidobacterium* closer to the definition of a foundation taxon (62) or that of an enterotype, which are dominant bacterial groups that can be used to classify individuals or microbiomes (63–65) and can be influenced by environmental factors (65, 66). In one such example of this, *Bifidobacterium*, *Bacteroides,* and *Prevotella* were found to be the dominant enterotypes in children and were used to find enterotype-specific correlation patterns related to diet (67).

Depletion of *Bifidobacterium* in combination with enrichment of *Bacteroides* and/or *Prevotella* has been observed in multiple studies of captive marmosets, often in response to a microbiome stressor (7, 10, 26, 68). This has led some to suggest that captivity “humanizes” the marmoset microbiome (10, 17, 69) since *Bacteroides* and *Prevotella* are typically the most prevalent genera in the human gut microbiome (17), while *Bifidobacterium* dominates wild marmoset microbiomes (6, 8). *Bifidobacterium*’s negative correlations within the *Bacteroidales* cluster (which includes *Bacteroides* and *Prevotella*) suggest a similar relationship in this study. One possible explanation for this is metabolic niche specialization. Bifidobacteria are believed to be an important source of carbohydrate-derived SCFAs in marmosets (8, 27), and some can specialize in degrading host-indigestible fibers (26). Because *Bacteroides* was associated with the SCFA isovalerate (Fig. 5B), which is primarily produced by proteolytic fermentation (70), it could potentially overtake carbohydrate-oriented bifidobacteria with increase in dietary protein intake (71). In combination with specialization, competition for overlapping niches could also occur (6). Some SCFA-producing *Bacteroidales* can also utilize dietary carbohydrates, for example (72, 73). Along these lines, overlapping bacteria-bacteria or bacteria-metabolite associations in this study might reflect similar functional roles within the microbiome (74–78). Such functional redundancy is to be expected based on similar results in humans and makes the gut microbiome more robust to perturbations (78, 79). In the event of such a disturbance, bacteria within the *Bacteroidales* cluster that share functionality with *Bifidobacterium* could potentially outcompete it and take its place as the predominant genus. Future studies will be needed to investigate this idea further.

Another potential source of competition is *Clostridium sensu stricto 1*, which had the highest KCS in this study. It was involved in the majority of significant bacteria-metabolite associations, indicating it could potentially play a gatekeeping role over metabolites involved in microbial growth and signaling. Of those, its associations with neuroactive metabolites glutamate and GABA are interesting because both can be produced/consumed by gut bacteria (80). Microbial involvement in these pathways was previously demonstrated in marmosets (40), suggesting that *Clostridium_sensu_stricto_1* could engage with the nervous system of its host. The fact that every differential metabolite *Clostridium_sensu_stricto_1* correlated with was taurine or a BA suggests it is also involved in tauroBA metabolism, which could subsequently modulate host BA synthesis via the farnesoid X receptor (52) or influence the growth of other taxa through multiple mechanisms (81). Correlation of *Clostridium_sensu_stricto* genera with BAs or BA-related genes has been reported before, though the correlation strength and specific metabolites differ between studies (82–87). The mutual correlation of taurocholic acid with *Clostridium_sensu_stricto_1* and *Olsenella* in this study could then affect bacteria in the *Bacteroidales* cluster (Fig.S7), several of which were previously linked to BA metabolism in marmosets (40, 88). In addition to being directly correlated to, and sharing common metabolites with, potentially pathogenic *Hafnia-Obesumbacterium* (89), *Clostridium_sensu_stricto_1* was found by one study to be enriched in marmosets with duodenal stricture (17). That study further proposed that *Clostridium perfringens* (one of several pathogenic species within the *Clostridium_sensu_stricto_1* genus [90, 91]) was a causative agent in stricture development in response to BA dysregulation. *Clostridium_sensu_stricto_1* can therefore be identified as a potential pathogen in marmosets and the strongest candidate keystone genus in this study.

The larger number of significant metabolite correlations (Fig. S2) and more pronounced temporal trend between metabolome samples (Fig. S3) suggest that metabolite abundance fluctuates more rapidly than bacterial abundance, in line with previous results in humans (43). The microbiome and metabolome can also change from host diet. Indeed, 5/7 BAs that were DA between Pre and Control samples were primary (i.e., host-synthesized), and a high-fat, low-fiber diet can increase BA synthesis in humans (92). Since the marmosets in this study were fed with a cafeteria-style diet (9), variation could be introduced from marmosets choosing to consume or ignore supplemental items served alongside their primary food source of canned Zupreem. However, the marmoset gut—at least in the wild—is fairly robust to changes in diet (13). While we concede the point that this study used captive marmosets, we note that gum arabic was supplemented into their diet, making it more similar to that of wild marmosets (26). Moreover, none of the significant changes in gut microbiome or metabolome abundance that were previously described in captive marmosets following a dietary transition (7, 10) were reciprocated here, suggesting that the impact of diet on our results was minimal.

This is the first study detailing longitudinal bacteria-bacteria associations between specific taxa in the marmoset gut microbiome. Metabolite-metabolite associations have been described for the marmoset serum metabolome (93), but to our knowledge, this is the first study to do so for the fecal metabolome. Although cross-comparison is limited by the former’s focus on phenylalanine, a significant methionine-phenylalanine-tyrosine association was reciprocated here. Simultaneous profiling of the serum and fecal metabolomes should be a goal of future marmoset studies to further elucidate the systemwide influence of gut microbiota. Our major findings in this study suggest that bifidobacteria are more abundant overall but could compete with other taxa like *Flavonifractor* and *Bacteroidales* members for dominance. Network analysis identified *Clostridium_sensu_stricto_1* as a particularly strong candidate keystone genus that could exert its influence by modifying BAs, allowing it to modulate the growth of neighboring bacteria and potentially affect intestinal integrity in its host (33). The KCS was developed to provide meaningful results in spite of study limitations, including a relatively small number of metabolites measured in a small number of samples, inference of microbial data at the genus level necessitated by the use of 16S sequencing, and the use of correlation-based analysis for compositional data, all of which limit data resolution. We acknowledge that central bacteria in a network are not necessarily keystones and vice versa (29). The KCS is therefore intended to function as a confidence index to identify the strongest candidate keystone taxa for more detailed follow-up analysis. While outside the scope of the current work, targeting the keystone candidates identified here could be an important future work needed to provide species-level accuracy and biological validation of our results. In future studies, we plan on enhancing the temporal resolution by including more subjects and time points, as well as introducing external manipulation to observe how gut microbiome compostion and function change in response to perturbation. This will allow higher-order network structures such as modules to be identified and provide greater confidence and specificity in identifying keystones.

## Data Availability

16S rDNA sequence data are available at the EMBL Nucleotide Sequence Data Base under the accession number PRJEB65103. Metabolomics data generated by this study are available at the National Institutes of Health (NIH) Common Fund’s National Metabolomics Data Repository (NMDR) website, the Metabolomics Workbench (94), assigned Study ID ST003530. The data can be accessed directly via Project (PR002172) DOI https://doi.org/10.21228/M8VV6J. Software, including the computer code, Cytoscape file, and custom Conda environment used during analysis, as well as the output files necessary to reproduce all figures and tables presented in this article, are available on GitHub at https://github.com/clayton-lab/marm_microbiome_metabolome_manuscript.

## SUPPLEMENTAL MATERIAL

File S1 msystems.00214-25-s0001.pdf Additional experimental details and supplemental figures and tables.

ASM does not own the copyrights to Supplemental Material that may be linked to, or accessed through, an article. The authors have granted ASM a non-exclusive, world-wide license to publish the Supplemental Material files. Please contact the corresponding author directly for reuse.
