# Supplementary material for: Microbiome and metabolome association network analysis identifies Clostridium_sensu_stricto_1 as a stronger keystone genus candidate than Bifidobacterium in the gut of common marmosets
Source: mSystems. 2025 Jul 7;10(8):e00214-25. doi: 10.1128/msystems.00214-25 (PMC12363189; doi:10.1128/msystems.00214-25)
Supplement: File S1 — Additional experimental details and supplemental figures and tables. [file msystems.00214-25-s0001.pdf]

Supplementary Methods

Additional experimental details from original study

The study utilized a crossover design (Figure 1; Figure S1) that was split into 3 repeating phases (Pre-Test, Test, Post-Test) with 2 treatment groups (G1, G2). While G1 marmosets were subjected to social isolation in the Test phase, G2 marmosets remained in their home cage. This switched half way through the study so that the G1 group remained in their home cages while the G2 group underwent isolation. No experimental manipulation occurred during the Pre-Test or Post-Test phases. During the social isolation (Iso) condition, marmosets were subjected to 5 hours of social isolation each day for 6 consecutive days. Marmosets placed in social isolation were moved to a wire mesh cage (60 × 60 × 60 cm) with access to food and water in a room with no conspecefics present. Testing ocurred at the same time each day, after which marmosets were returned to their home cage. Fecal samples were collected from each marmoset in their home cage one hour before testing occurred.

Subject metadata including the sexes and ages of each marmoset sampled in this study is provided in Table S1. Prior to the study, subjects were inspected by a Doctor of Veterinary Medicine and determined to be free from complications that would disquality them from being classified as clinically healthy. Subjects were housed at the University of Nebraska at Omaha's Callitrichid Research Center in varying group sizes ranging from 2-9 individuals per cage, with 4-6 cages per room. Each room was kept at an ambient temperature range between 19-22°C with an automated 12-hour light:dark cycle. Cages (0.76 × 1.63 × 1 m) were made of wire mesh with wood shavings lining the floor, and contained shelves, cloth hammocks, and enrichment objects such as branches and tubes. Marmosets were fed a daily diet of canned Zupreem supplemented with various other foodstuffs including fried eggs, fruits and vegetables, mealworms, applesauce enriched with calcium and vitamin C, and Mazuri gum arabic. Food bowls and water bottles were refilled at approximately the same time each day. Marmosets also received miniature marshmallow pieces as rewards to facilitate sample collection.

Sample extraction and metabolite quantitation for short-chain fatty acids and bile acids

1. Short chain fatty acids (SCFAs) assay using GC-MS: An aliquot of 50 mg of fecal sample was extracted using 0.5% phosphoric acid spiked with 83.7 µg of D3-acetate as the internal standard. The samples were disrupted and homogenized by adding 2 stainless steel beads (SSB 32) using the TissueLyserII at 20 Hz for 2 min. The samples were additionally sonicated for 5 min. After centrifugation at 16,000 g for 10 min, the supernatants were transferred to a new tube. Butanol was added to the supernatant, and samples were extracted one more time using the TissueLyserII at 2 Hz for 2 min. The samples were centrifuged at 16,000 g for 10 min and the upper phase was transferred to a new tube. A small aliquot of each sample was pooled together to make a quality control (QC) sample. The samples were transferred to GC vials and injected without derivatization. The GC-MS analysis was carried out using the same equipment described in the untargeted metabolomics section. The injector temperature was 250°C; the MS transfer line was 230°C. Metabolites were separated on a VF-WAXms column (30 m x 0.25 mm, 0.25 µm, Agilent Technologies), at constant flow of 1.2 $ml.min^{-1}$ of helium as a carrier gas. One microliter of sample was injected into the injector operating in 1:2 split mode. The temperature of the column was initially set to 70°C, and increased at a rate of 12°C.min^{-1} to 170°C, and then at 25°C.min^{-1} to 250°C, followed by a hold for 10 min. The acquisition was set up as a SIM (Single Ion Monitoring) scan method using selected ions to analyze the detectable SCFAs (D3-acetate, 46-63 ions; acetate, 43-60 ions; propionate, 45-74 ions; butyric acid, 60-73 ions; isovaleric acid, 60-74 ions; valeric acid, 60-73 ions). The data was acquired at a scan speed of 3.125 u/s with a dwell time of 30 ms for each ion selected. The generated data was analyzed with Agilent Mass Hunter Quantitative Analysis. For quantification, an external standard curve was prepared using a series of standard samples containing different concentrations of SCFAs and fixed concentration of the internal standard.

2. Bile acids assay using LC-MS/MS: An aliquot of 50 mg of fecal samples was extracted by adding 2 stainless steel beads (SSB 32) and chilled methanol:acetonitrile (1:1) solution using the TissueLyserII at 20 Hz for 3 min. The internal standard used is a mixture of several isotope labelled bile acids (D4-taurochenodeoxycholic acid; D4-taurocholic acid;

D4-glycocholic acid; D4-glycochenodeoxycholic acid; D4-chenodeoxycholic acid; D4-deoxycholic acid). Samples were centrifuged at 4°C at 16,000 g for 10 min, and supernatants were transferred to new tubes. Samples were extracted the same way a second time with supernatants combined to the first one and then dried down using a SAVANT speed-vac. Pellets were resuspended using 30% methanol and transferred to HPLC vials. The samples were analyzed by LC-MS/MS using Multiple Reaction Monitoring (MRM) scan mode. The UPLC Nexera X2 (Shimadzu, Columbia, MD, USA) system used was interfaced with a QTRAP 6500+ (Sciex, Redwood City, CA, USA) mass spectrometer equipped with a TurboIonSpray (TIS) electrospray ion source. Analyst software (version 1.6.3) was used to control sample injection, separation, acquisition and data analysis. Bile acids were separated using the ACCQ-TAG ULTRA C18 (2.1 x 100 mm, 1.7 µm, Waters) running at a flow rate of 0.4 mL/min. The gradient of the mobile phases A (2 mM ammonium formate/0.5% formic acid) and B (0.5% formic acid/10% isopropanol/90% acetonitrile) was as follows: 30% B for 1.5 min, to 55% B in 0.2 min, to 98% B in 3.3 min, hold at 98% B for 5 min, to 30% B in 0.5 min. The column compartment was set at 55°C. The QTRAP 6500+ mass spectrometer was tuned and calibrated according to the manufacturer's recommendations. The mass spectrometer was operated with the IonDrive Turbo V electrospray ionization (ESI) source in negative ion mode. The ESI source operation parameters were as follows: source temperature at 400°C; ion spray voltage at –4500; ion source gas 1 at 40; ion source gas 2 at 40; curtain gas at 20 psi; collision gas at medium. The bile acids were detected using MRM transitions that were optimized using standards. The MRM transition (Q1-Q3) for the compounds are as follow: α-muricholic acid (407-387), β-muricholic acid (407-371), chenodeoxycholic acid (391-373), cholic acid (407-343), deoxycholic acid (391-345), glycochenodeoxylic acid (448-74), glycocholic acid (464-74), glycodeoxycholic acid (448-74), glycolithocholic acid (432-74), hyocholic acid (407-389), lithocholic acid (375-357), ω-muricholic acid (407-387), taurochenodeoxycholic acid (498-80), taurocholic acid (514-80), taurodeoxycholic acid (498-80), taurolithocholic acid (482-80). For quantification, an external standard curve was prepared using a series of standard samples containing different concentrations of unlabeled compounds and fixed concentrations of the internal standards.

Power-law fitting to network graph degree distributions

Power-law fitting to network graph degree distributions Network graphs of metabolite-metabolite, bacteria-metabolite, and bacteria-bacteria associations were exported from Cytoscape in CSV format, and their degree distributions were calculated and plotted in R. For each degree distribution, a power-law distribution was fitted and goodness of fit was assessed with the poweRlaw package. This package was also used to calculate the significance of the fit by performing a Kolmogorov-Smirnov test using 5000 bootstrap iterations, and to compare the fit of the power-law distribution to those of the log-normal, exponential, and poisson distributions using Vuong's method. The output of the poweRlaw package can be found in the supplementary results under the heading "Power-law fitting to network graph degree distributions". The bootstrap goodness-of-fit p-value tests the null hypothesis that the fitted data comes from a power-law distribution, meaning that significance ($p<0.05$) indicates a poor fit. Vuong's test statistic is used to calculate a p-value for both a one-sided and two-sided test. The one-sided p-value is order-dependent, and tests if fit1 (the power-law fit in this case) is significantly better than fit2. The two-sided p-value does not depend on order, and tests if either fit1 or fit2 is significantly better than the other.

Supplementary Results

LEfSe output (differential genera abundance) by treatment condition

```
Number of significantly discriminative features: 1 ( 1 ) before internal wilcoxon
Number of discriminative features with abs LDA score > 2.0 : 0
No differentially abundant features found in
/path/to/lefse/results/relative_genera_lefse.res
```

Power-law fitting to network graph degree distributions

```
###################################

```

```
metabolite-metabolite power-law bootstrap

##################################
$p:
0.0486

$goodness_of_fit:
0.156812326960764

$sim_time:
0.0950726165771484

$seed:
777

$package_version:
0.70.6

$distance:
ks

########################

power-law fit compared to other distributions

########################
----------------------
log-normal comparison
----------------------
$test_statistic:
-1.31438154218327
```

```
$p_one_sided:
0.905641080616967

$p_two_sided:
0.188717838766067

----------------------
exponential comparison
----------------------
$test_statistic:
-2.56412131161116

$p_one_sided:
0.994828130770046

$p_two_sided:
0.0103437384599074

----------------------
poisson comparison
----------------------
$test_statistic:
-1.6563972647142

$p_one_sided:
0.951179302666206

$p_two_sided:
0.0976413946675881
```

```
##################################

bacteria-bacteria power-law bootstrap

##################################
$p:
0.1378

$goodness_of_fit:
0.113300036846848

$sim_time:
0.0534451133728027

$seed:
777

$package_version:
0.70.6

$distance:
ks

########################

power-law fit compared to other distributions

########################
----------------------
log-normal comparison
----------------------
```

```
$test_statistic:
-1.38639353709692

$p_one_sided:
0.917186615583154

$p_two_sided:
0.165626768833692

----------------------
exponential comparison
----------------------
$test_statistic:
-1.24443307745466

$p_one_sided:
0.893329892324806

$p_two_sided:
0.213340215350389

----------------------
poisson comparison
----------------------
$test_statistic:
1.49118672515662

$p_one_sided:
0.0679562389651349

$p_two_sided:
```

```
0.13591247793027

##################################

bacteria-metabolite power-law bootstrap

##################################
$p:
0.5408

$goodness_of_fit:
0.0583183281903178

$sim_time:
0.0572966812133789

$seed:
777

$package_version:
0.70.6

$distance:
ks

########################

power-law fit compared to other distributions

########################
----------------------
```

```
log-normal comparison
----------------------
$test_statistic:
-0.447887683462267

$p_one_sided:
0.672882871157588

$p_two_sided:
0.654234257684823

----------------------
exponential comparison
----------------------
$test_statistic:
1.01059712048174

$p_one_sided:
0.156104647287584

$p_two_sided:
0.312209294575168

----------------------
poisson comparison
----------------------
$test_statistic:
1.3008322856573

$p_one_sided:
0.0966579341124696
```

```
$p_two_sided:
0.193315868224939
```

Supplementary Tables and Figures

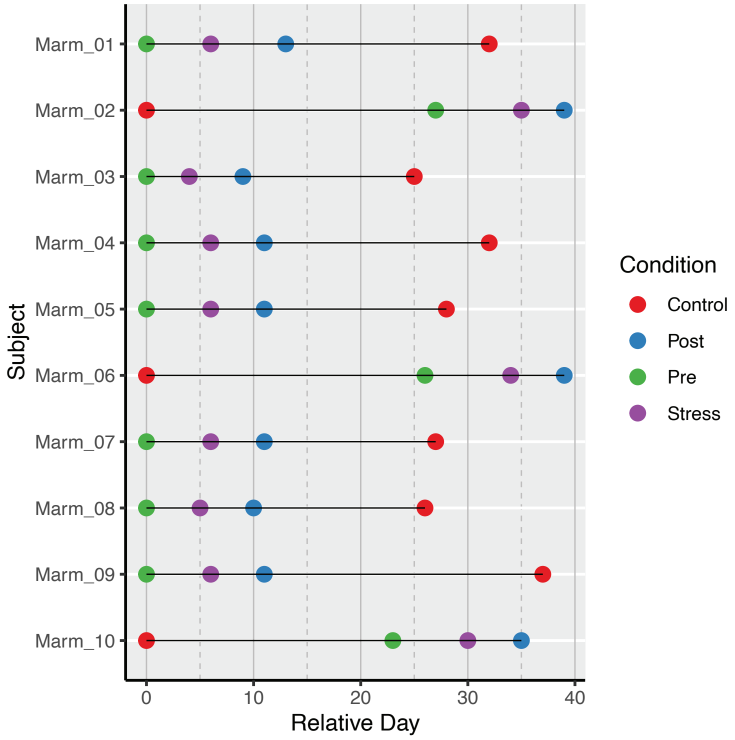

FIG S1 Sample collection timeline. Individual subjects are displayed along the Y axis, while the X axis denotes the number of days relative to the first sample collected (relative day 0).

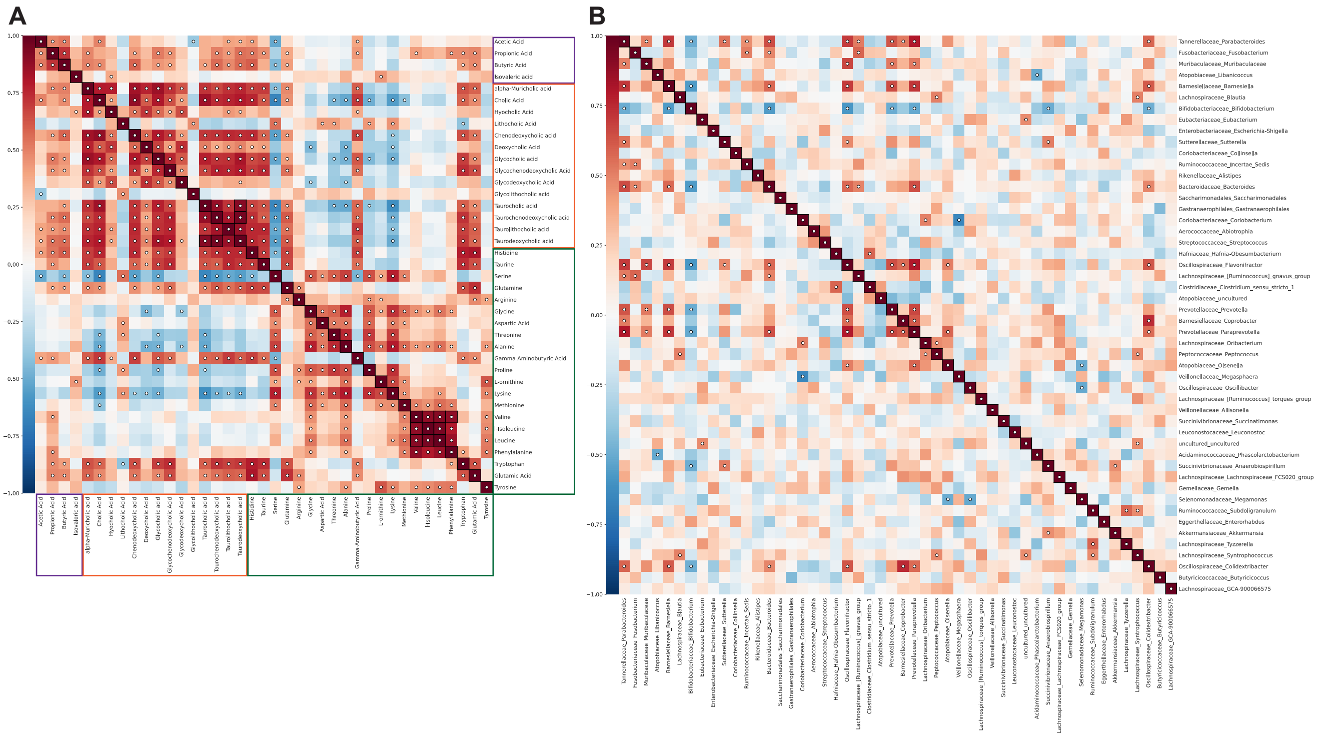

FIG S2 Metabolites exhibit stronger and more numerous within-dataset correlations than microbial genera. Heatmaps depicting within-dataset Spearman correlation coefficient for (A) metabolomics and (B) relative genera abundance profiles. White dots depict associations with marginal FDR-adjusted p-value < 0.05. Colored squares surrounding text in (A) denote metabolite panel membership, with purple, orange, and green corresponding to targeted panels for short-chain fatty acids, bile acids, and amino acids, respectively.

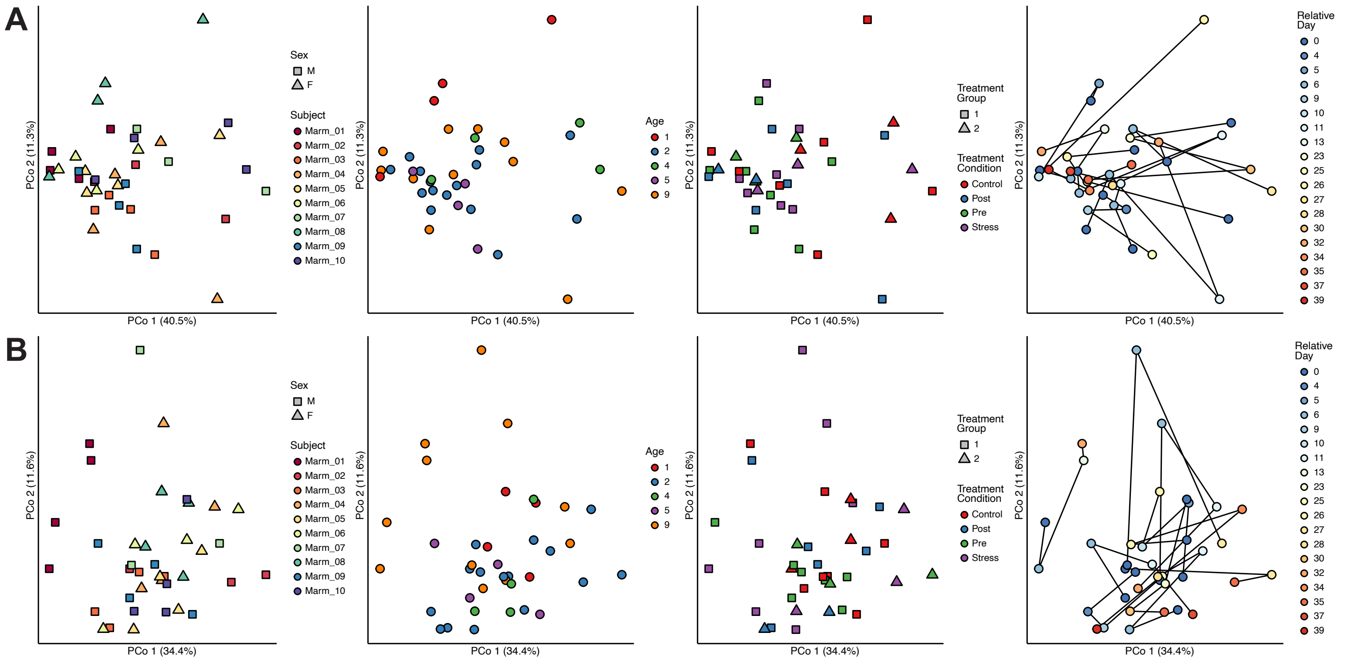

FIG S3 Gut microbial and metabolite abundance diverges from baseline (relative day 0) over a period of 40 days. Principal coordinates plot of Bray-Curtis dissimilarity for (A) metabolomics and (B) relative genera abundance profiles. Two samples (Marm_02 Relative_Day 39 and Marm_07 Relative_Day 11) were excluded from B due to low total read abundance.

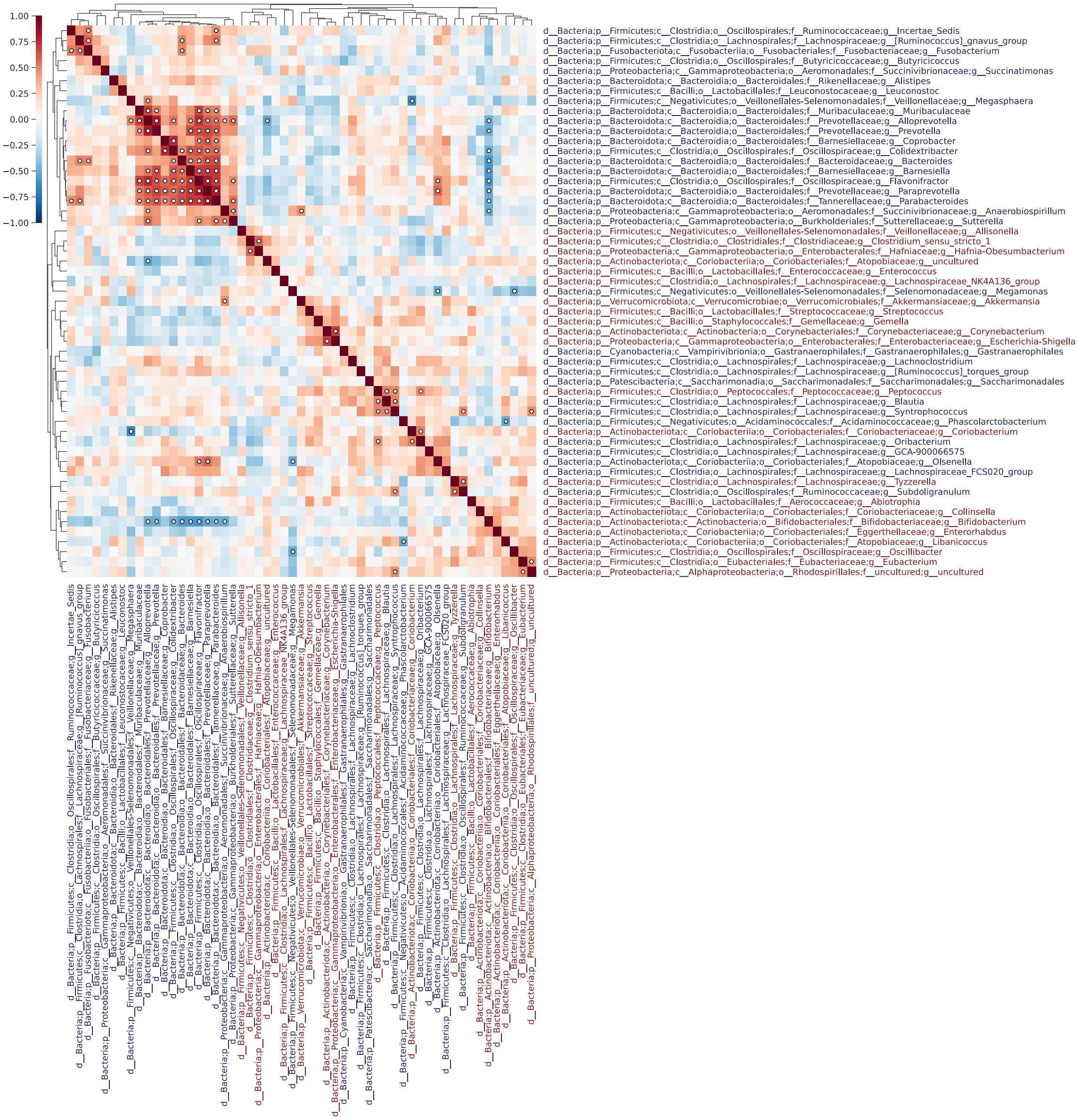

FIG S4 Identical clustermap as the one depicted in Figure 2B, but with the full taxonomy for each ASV.

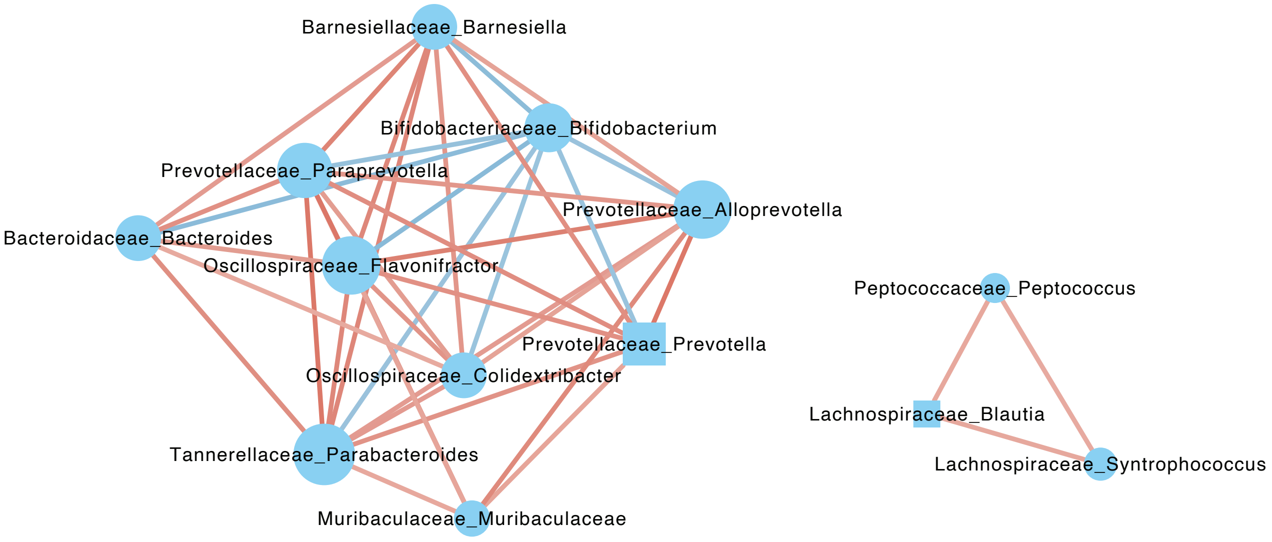

FIG S5 Clusters found by the MCODE plugin in the bacteria-bacteria subgraph. Nodes with the same color are members of a cluster, and square nodes are "seed nodes" around which each cluster was built.

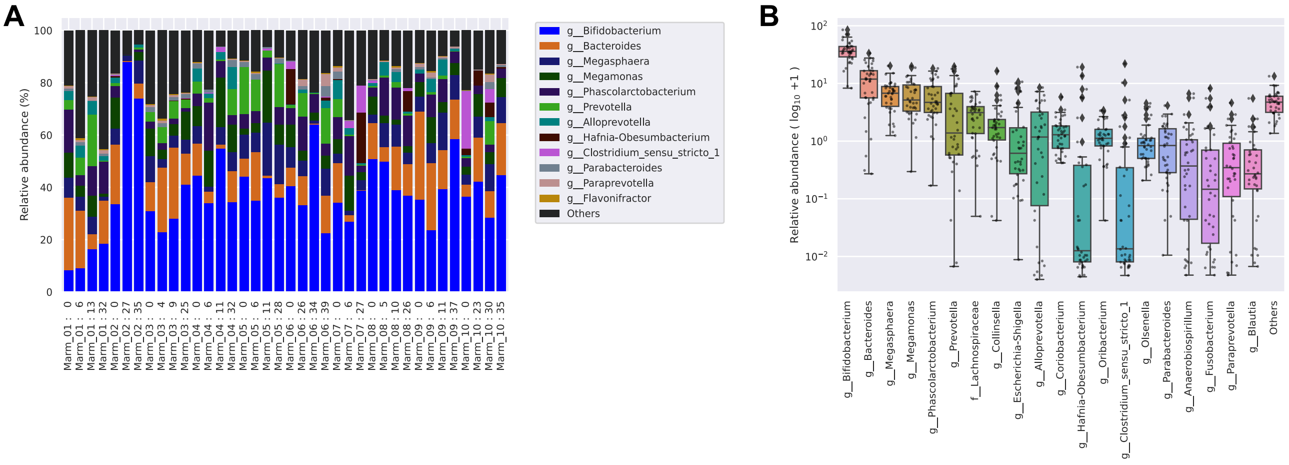

FIG S6 *Bifidobacterium* was the dominant genus for all but 1 subject in this study. (A) Relative abundances of the 6 most abundant and the 6 most central genera as measured by 16S amplicon sequencing. X-axis labels correspond to the subject ID and relative day of each sample, separated by a colon. (B) Boxplot showing distribution of 16S amplicon relative abundance for each sample. Taxa are ordered by descending mean abundance. Abundance is denoted along the y-axis on a log-scale, with a pseudocount of 1 added to remove zeros. Median abundance is denoted by horizontal lines within each box.

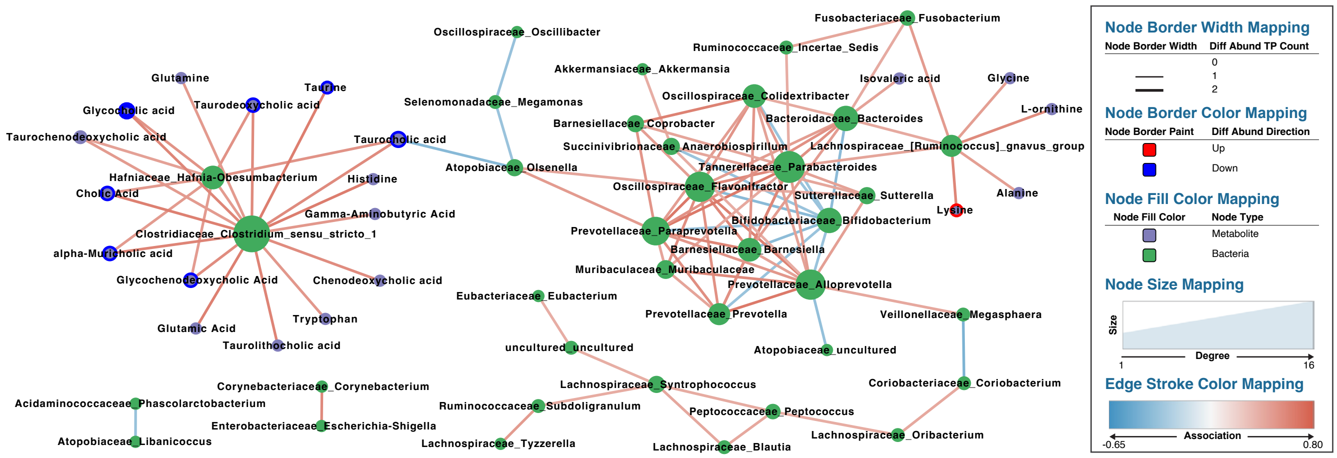

FIG S7 Bacteria-metabolite and bacteria-bacteria network graphs from Figure 5 combined into a single pane. This makes it easier to visualize bacteria that were simultaneously involved in bacteria-bacteria and bacteria-metabolite associations. Green nodes are bacterial genera and purple nodes are metabolites. Node size corresponds to degree, while edge color corresponds to the sign and strength of associations. Differentially abundant metabolites are denoted by colored node borders, with the thickness of each border denoting the number of experimental phases in which the abundance change was significant.

TABLE S1 Subject metadata for the marmosets included in this study.

Subject ID	Treatment Group	Sex	Cage	Age	Health Status
Marm_01	1	M	C3	9	Healthy
Marm_02	2	M	C4	2	Healthy
Marm_03	1	M	A5	2	Healthy
Marm_04	1	F	G3	9	Healthy
Marm_05	1	F	C1	2	Healthy
Marm_06	2	F	C3	2	Healthy
Marm_07	1	M	G3	9	Healthy
Marm_08	1	F	G2	1	Healthy
Marm_09	1	M	G2	5	Healthy
Marm_10	2	M	G4	4	Healthy

TABLE S2 Network densities for association network subgraphs.[a]

Subgraph Name	Network Density	Network Heterogeity
Metabolite-Metabolite	0.371	0.432
Amino Acid-Amino Acid	0.376	0.298
Bile Acid-Bile Acid	0.924	0.105
Bacteria-Metabolite	0.15	1.299
Bacteria-Bacteria	0.153	0.793

[a]Network densities for the metabolite-metabolite (including each panel separately), bacteria-bacteria, and bacteria-metabolite subgraphs. Network densities for the metabolite panel-specific subgraphs were only calculated for metabolite-metabolite associations to directly compare overall metabolite-metabolite and microbe-microbe connection densities between subgraphs. The SCFA-SCFA network is not included in this table because the network was too small to calculate statistics for.
